# Integrative medicine (East Asian herbal medicine combined with conventional medicine) for psoriasis: A protocol for systematic review and meta-analysis

**DOI:** 10.1097/MD.0000000000032360

**Published:** 2023-01-20

**Authors:** Hyehwa Kim, Hee-Geun Jo, Ji-Hye Hwang, Donghun Lee

**Affiliations:** a Department of Herbal Pharmacology, College of Korean Medicine, Gachon University, Sujeong-gu, Seongnam, Republic of Korea; b Naturalis Inc. 6, Daewangpangyo-ro, Bundang-gu, Seongnam, Gyeonggi-do, Republic of Korea; c Department of Acupuncture and Moxibustion Medicine, College of Korean Medicine, Gachon University, Sujeong-gu, Seongnam, Republic of Korea.

**Keywords:** herbal medicine, integrative medicine, meta-analysis, psoriasis, systematic review

## Abstract

Psoriasis is a chronic, inflammatory, autoimmune skin disease. The aim of this review is to systematically evaluate the efficacy and safety of integrative medicine (East Asian herbal medicine combined with conventional medicine) used to treat inflammatory skin lesions of psoriasis.

**Methods:**

A comprehensive literature search will be conducted in 3 English databases (PubMed, Cochrane Library, and Embase), 4 Korean databases (Korean Studies Information Service System, Research Information Service System, Oriental Medicine Advanced Searching Integrated System, and Korea Citation Index), 2 Chinese databases (Chinese National Knowledge Infrastructure Database and Wanfang data), and 1 Japanese database (Citation Information by National Institute of Informatics) for randomized controlled trials from their inception until July 29, 2021. Statistical analysis will be performed using *R* version 4.1.2 and the R studio program using the default settings of the “meta” and “metafor” packages. The primary outcome will be an improvement in the psoriasis area severity index. All outcomes will be analyzed using a random-effects model to produce more statistically conservative results. If heterogeneity is detected in the study, the cause will be identified through sensitivity, meta-regression, and subgroup analyses. Methodological quality will be assessed independently using the revised tool for the risk of bias in randomized trials, version 2.0. The overall quality of evidence will be evaluated according to the Grading of Recommendations Assessment, Development, and Evaluation pro framework.

**Results:**

This study will review all available trials on the same subject and arrive at a more statistically robust conclusion based on a sufficient sample size of participants and additional analysis using data mining techniques will be performed on intervention prescription information in clinical studies collected according to rigorous criteria.

**Conclusion:**

We believe that this study will provide useful knowledge on managing inflammatory skin lesions of psoriasis vulgaris using integrative medicine using East Asian herbal medicine.

**Key points**

- This study will only consider randomized controlled clinical trials of East Asian medicinal herb as an integrative medicine, not monotherapy.
- Sensitivity analysis, meta-regression, and subgroup analysis will all be used to derive more rigorous evidence.
- Among the various conditions of psoriasis, the effectiveness of integrative medicine for inflammatory skin lesions will be reviewed.
- Searches will be conducted without restrictions on language, nationality or race.
- Based on additional analysis of the collected drug data, additional pharmacological information to support clinical effects will be derived.

## 1. Introduction

Psoriasis is a chronic inflammatory skin disease with various clinical manifestations affecting millions of individuals worldwide.^[1]^ The incidence of psoriasis has been investigated in various countries. A 2013 global epidemiology-related study reported a prevalence of 0.91% to 8.5% in adults, and later, a Danish cohort study found that the lifetime prevalence diagnosed by doctors was 6.3%.^[2,3]^ Chronic inflammation caused by uncontrolled keratinocyte proliferation and differentiation is a hallmark of this disease.^[4]^ Inflammatory skin symptoms on exposed body parts, such as the face and limbs, severely adversely affect the daily life of most patients with psoriasis.^[5]^ Many patients with chronic psoriasis develop various complications that can shorten their life expectancy.^[6,7]^ Therefore, researching a solution to control the serious influence of psoriasis on physical, social, and psychological welfare through active treatment is currently required.

Clinically, psoriasis can be classified as erythrodermic psoriasis, guttate psoriasis, pustular psoriasis, and psoriasis vulgaris.^[1]^ Most psoriasis lesions are skin-related, with psoriasis vulgaris accounting for approximately 90% of all cases.^[1,4,6]^ Individuals with psoriasis often require lifelong treatment because psoriasis is a long-lasting disease requiring prolonged use of medications. Therefore, all treatment regimens must meet the stringent patient safety requirements. Even if there are several conventional medications (CMs) in the market, systemic therapy for psoriasis still has certain issues that need to be resolved. For instance, acitretin is contraindicated in women of reproductive age because of its teratogenicity, and adverse events (AEs) such as dose-dependent hair loss and xerosis have been reported.^[4,8]^ Meanwhile, methotrexate, which has been used for a long time, also has adverse effects, such as hepatotoxicity and bone marrow suppression, which can lead to cirrhosis.^[1,9]^ Moreover, the cost of these drugs is a significant factor that lowers adherence to treatment and accessibility. In this context, in relation to the treatment of psoriasis, an extensive search for new drug candidates that are more cost-effective, safe, and effective than CMs must be undertaken.

Natural products are regarded as promising candidates for various chronic diseases worldwide because they are safer than new compounds even after long-term use, with high patient compliance.^[10,11]^ Among these trends, the most active search for candidate materials linked to psoriasis is taking place in East Asian herbal medicine (EAHM).^[12–16]^ “EAHM” refers to herbal remedies that are used as medicines to treat illnesses in several East Asian nations, including Korea, China, Taiwan, and Japan.^[12–15,17–20]^ On the other hand, studies on chronic disease management applying the integrative medicine (IM) perspective tend to be actively conducted in countries with experience in using medicinal herbs.^[21–28]^ IM is a holistic approach that simultaneously utilizes all available complementary treatments and conventional treatments.^[29]^ The combination of CMs and herbal remedies, in particular, as a subsection of IM, has demonstrated better therapeutic performance and safety than conventional treatments in various conditions, including coronavirus disease 2019, cancer, stroke, chronic pruritus, and rheumatoid arthritis.^[21,25,30–34]^ Based on several previous studies related to this topic, it can be hypothesized that IM potentially improves the psoriasis area and severity index (PASI) and clinical symptoms and reduce AEs associated with CMs in patients with psoriasis.^[35–39]^

Based on previous study knowledge and awareness, the authors set the following research objectives: Through a systematic review of randomized controlled clinical trials (RCTs), it was confirmed whether IM using EAHM is worth exploring as a useful candidate for inflammatory skin lesions in psoriasis vulgaris, thereby providing information to assist decision-making, and; Additional data analysis of the herbal prescription data collected through this review will lead to hypotheses related to promising candidates for optimal IM for psoriasis vulgaris.

## 2. Methods

### 2.1. Protocol registration

This review will be conducted in accordance with the preferred reporting items for systematic reviews and meta-analysis (PRISMA) 2020 statement.^[40]^ The protocol of this systematic review was prepared according to PRISMA Protocols 2015^[41]^ and was pre-registered in PROSPERO (registration number: CRD 42022296852, available from https://www.crd.york.ac.uk/PROSPERO/display_record.php?RecordID=296852).

### 2.2. Search strategy

**Figure 1.**
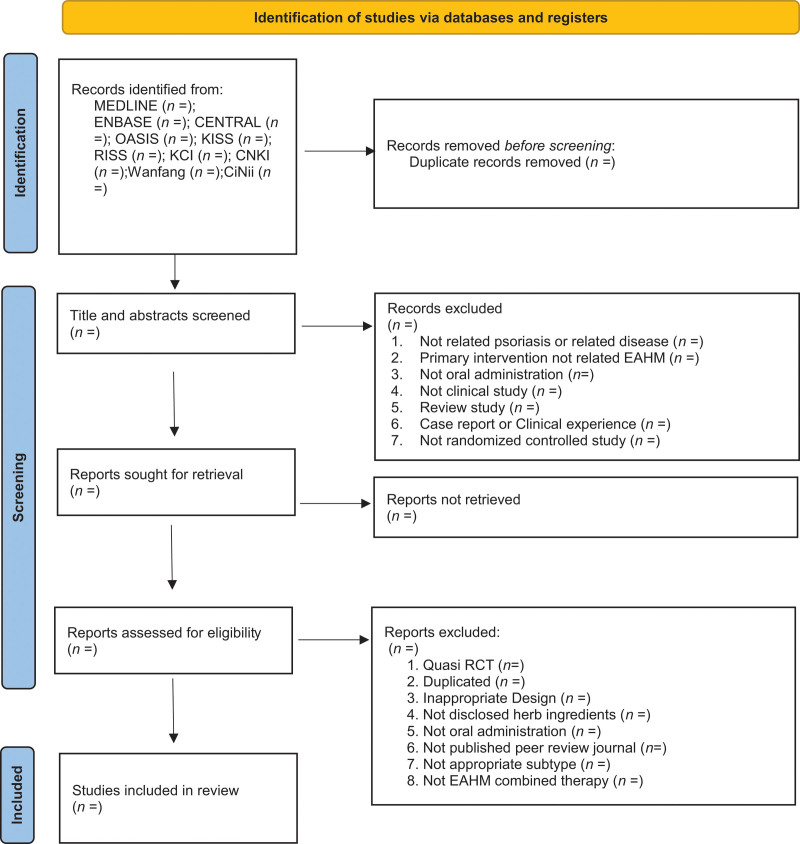
Preferred reporting items for systematic reviews and meta-analysis 2020 flow diagram.

RCTs that evaluated the efficacy and safety of IM for psoriasis vulgaris will be searched in the following 10 electronic databases from their inception until July 29, 2021: 3 English databases (PubMed, Cochrane Library, and Embase), 4 Korean databases (Korean Studies Information Service System, Research Information Service System, Oriental Medicine Advanced Searching Integrated System, and Korea Citation Index), 2 Chinese databases (Chinese National Knowledge Infrastructure Database, Wanfang data), and 1 Japanese database (Citation Information by National Institute of Informatics). The following Boolean format will be used for the search: (Psoriasis [Mesh]) AND [(Psoriasis [Title/Abstract]) OR (Pustulosis of Palms [Title/Abstract] AND Soles [Title/Abstract]) OR (Pustulosis Palmaris et Plantaris [Title/Abstract]) OR (Palmoplantaris Pustulosis [Title/Abstract]) OR (Pustular Psoriasis of Palms [Title/Abstract] AND Soles [Title/Abstract])] AND (“Plants, Medicinal”[MeSH] OR “Drugs, Chinese Herbal”[MeSH] OR “Medicine, Chinese Traditional”[MeSH] OR “Medicine, Kampo”[MeSH] OR “Medicine, Korean Traditional”[MeSH] OR “Herbal Medicine”[MeSH] OR “Prescription Drugs”[MeSH] OR “traditional Korean medicine”[Title/abstract] OR “traditional Chinese medicine”[Title/abstract] OR “traditional oriental medicine”[Title/abstract] OR “Kampo medicine”[Title/abstract] OR herb* [Title/abstract] OR decoction* [Title/abstract] OR botanic* [Title/abstract]). These search terms will be appropriately modified to search the Korean, Chinese, and Japanese databases. The PRISMA 2020 flow chart will illustrate the number of articles identified, screened, included, and excluded (Fig 1).

### 2.3. Study selection

#### 2.3.1. Type of studies.

Only RCTs evaluating the efficacy and safety of IM for plaque psoriasis will be included. There will be no restrictions on the language or publication time. Studies that meet the following criteria will be excluded:

(a) studies that are not RCTs or quasi RCTs.
(b) studies not related to plaque psoriasis or related diseases.
(c) primary intervention not related to IM.
(d) no oral administration of medications.
(e) not a clinical trial.
(f) case reports or reviews.
(g) studies not published in scientific peer-reviewed journals, including postgraduate theses or dissertations, and.
(h) studies in which the experimental intervention was not based on an IM approach, such as EAHM monotherapy.

#### 2.3.2. Type of participants.

Trials will be eligible for inclusion if they were conducted in patients with a diagnosis of psoriasis vulgaris, with no restrictions on age, sex, or race. Only studies for which formal or objective diagnostic criteria have been presented will be considered. Since the subject of this review was plaque psoriasis, clinical trials that included patients with other subtypes of psoriasis, such as psoriatic arthritis, guttate psoriasis, palmoplantar pulposus, and erythrodermic psoriasis, will be excluded from the review.

#### 2.3.3. Type of interventions.

RCTs that compared IM (EAHM combined with CMs) as an active intervention in the treatment group versus CMs alone in the control group will be included. RCTs that used IM as an intervention, on the other hand, will be regarded beyond the scope of the review and will be omitted. All dosage forms of IM intervention, such as decoction, granules, capsules, and pills, for inflammatory skin lesions in psoriasis, will be considered. There will be no restrictions on the dose and duration of treatment; however, the mode of delivery will be limited to oral intake. Studies wherein other complementary interventions, such as acupuncture, massage, or non-drug therapy, were only combined in the experimental group will be excluded. Meanwhile, studies that were unable to verify the composition of specific herbal constituents that comprised the EAHM prescription will be omitted.

#### 2.3.4. Type of outcome measures.

The response rates of patients with an improved PASI of 60% will be adopted as the primary endpoint. The absolute difference in PASI scores between the groups will also be used as the primary outcome. PASI 70, recurrence rate, Tumor necrosis factor-alpha, dermatology life quality index, interleukin-8(IL-8), IL-17, IL-22, and IL-23, interferon gamma (INF-*γ*) will be included as secondary outcomes. In addition, the incidence of AEs will be included in the secondary outcome to evaluate the safety of the intervention in patients with psoriasis.

### 2.4. Data extraction and management

The titles and abstracts of potentially eligible studies will be independently screened by 2 investigators (HK and HGJ) according to the above-mentioned search strategy. Subsequently, a full-text review will be performed based on the inclusion and exclusion criteria. Information on the included studies will be extracted independently by 2 reviewers (HK and HGJ). For the selected studies, 2 reviewers will independently collect the following information:

1. Publication information (title, first author, year of publication, and funding source).
2. Study characteristics (trial design, randomization method, sample size, treatment duration, and morbidity period).
3. Participants (age, sex, diagnostic criteria, and number of participants in each group).
4. Intervention (experimental intervention, comparator, ingredients, and detailed information on intervention frequency of medication, dosage, mode of delivery, and course of treatment).
5. Outcomes (primary and secondary outcomes, measurement point, blinding of outcome assessment, and AEs).

Any discrepancies will be resolved through discussions among the researchers. We will contact the authors of the included trial if more information is required.

### 2.5. Methodological quality assessment

The methodological quality of each included study will be independently evaluated by 2 investigators according to the risk of bias in randomized trials, version 2.0.^[42]^ Risk of bias in randomized trials 2.0 comprises 5 domains: bias arising from the randomization process, bias deviating from the intended intervention, bias due to the omission of outcome data, and bias in the selection of reported outcomes. The methodological quality is “high risk of bias,” it will be assessed on 3 levels of “low risk of bias” and “some concerns,” and discrepancies between raters will be resolved through discussion.

### 2.6. Statistical analysis

#### 2.6.1. Evidence synthesis.

Evidence synthesis of included studies with available data will be performed by calculating the effect size and 95% confidence intervals (CIs) using only the random-effects model. Heterogeneity will be considered statistically significant when the *P*-value based on the *χ*^2^ test is < 0.10 or *I*² is ≥ 50%. Statistical significance will be set at *P* < .05. Statistical synthesis of individual research results will be performed using the software R version 4.1.2 and R studio program (Version 1.4.1106, Integrated Development for R. RStudio, PBC, Boston, MA) using the default settings of the “meta” package and “metafor” package.^[43]^ In this review, to effectively reveal the exact value of the effect size without relying only on the *P* < .05 significance threshold in interpreting the primary outcome synthesis result, a drapery plot will be additionally illustrated along with the forest plot.^[44]^ The studies will be grouped according to the type of intervention, such as IM and comparator CM type. The relative risk and 95% CI will be calculated for PASI 60, PASI 70, and recurrence rate. The mean difference and 95% CIs will be calculated for the continuous PASI score and dermatology life quality index. For tumor necrosis factor-alpha, IL-8, IL-17, IL-22, IL-23, INF-*γ*, the standardized mean difference and 95% CIs will be calculated to integrate the results of several types of indicators for the same measurement target. AEs will be calculated using the odds ratio because the probability of the occurrence of an event is significantly lower than that of other outcomes, and it is necessary to estimate a causal relationship.

#### 2.6.2. Identifying the cause of heterogeneity.

In the meta-analysis results, if heterogeneity is confirmed in an primary outcome that synthesized the results of more than 10 trials, the following additional analysis will be performed to determine the cause. First, a sensitivity analysis will be performed according to the leave-1-out method to determine whether there was an effect of outliers in the included data. If no outliers are identified, after performing meta-regression analysis for the following 8 items specified in advance variables:

(i) Type of comparator.
(ii) Diagnostic criteria.
(iii) Treatment duration.
(iv) Source of the investigational medication.
(v) Type of preparation.
(vi) Number of participants.
(vii) Randomization method.
(viii) Risk of bias - which causes a significant difference in results, a subgroup analysis will be performed.

#### 2.6.3. Assessment of publication biases.

To distinguish publication bias, a contour-enhanced funnel plot will be used for the outcome of more than 10 trials in the meta-analysis.^[45]^ For the asymmetry of the visually confirmed funnel plot, Egger’s test and Begg’s test will be additionally performed to specifically confirm the existence of publication bias.^[46,47]^

#### 2.6.4. Quality of evidence according to outcome measures.

The overall quality of evidence for each outcome will be evaluated using the grading of recommendations assessment, development, and evaluation pro framework,^[48]^ grading of recommendations assessment, development, and evaluation evaluates the overall quality of evidence at 4 levels: very low, low, moderate, and high. The level of evidence is lowered according to factors such as the risk of bias, inconsistency, indirectness, imprecision, and publication bias.

### 2.7. Further analysis

If IM is significantly superior to the control group for inflammatory skin lesions, we will consider it as the base data for useful candidate materials. Accordingly, additional analysis will be performed on the already collected IM formulation data to identify important materials, with the frequency of use of individual materials, connectivity between materials, and centrality using unsupervised learning techniques, including association rule analysis and social network analysis. Finally, we will use network pharmacology techniques to identify key candidate materials to predict whether their mechanism of action supports clinical effects and what synergistic effects will be exerted through the connection between materials.

### 2.8. Amendments

The specifics and dates of any revisions will be noted in the final report if the protocol is changed substantially or modified.

### 2.9. Ethics and dissemination

Personal information will not be disclosed or published during the implementation of this systematic review. This review will not infringe on the rights of participants. Since it is not a clinical study that directly recruits participants, ethical approval is not required. The results of this study will be reported in a peer-reviewed scientific journal.

## 3. Discussion

This systematic review aims to evaluate the efficacy and safety of IM using EAHM for psoriasis vulgaris based on the maximum searchable trial range and derive practical drug development-related hypotheses.

Previous studies on similar subjects encountered challenges in deriving meaningful knowledge.^[35,36,38,49]^ First, although it has been demonstrated several times that IM is more effective than other controls, a more definite conclusion could not be drawn due to the heterogeneity between studies and too small sample size. Second, as explained in the introduction, EAHM is used as an individually customized polyherbal formulation according to the target indication. Therefore, the dosage and composition of IM interventions used in most clinical trials are heterogeneous. This is considered to be the major cause of the heterogeneity observed in the preceding meta-analysis. In addition, this characteristic makes it challenging for readers to determine whether the formula in a specific trial is related to the optimal IM. Furthermore, these factors make it challenging to derive hypotheses for follow-up studies, impairing the possibility of using the research. Third, the above intervention is a complex mixture comprising several different components, and the synergistic activity induced here is expected. In other words, an in-depth analysis of the link between the indications and the mode of mechanism that can be confirmed in experimental interventions will be needed to determine the optimal IM strategy.^[50–52]^

With this recognition, we expect to achieve the following goals: Potentially, we will review all available trials on the same subject and arrive at a more statistically robust conclusion based on a sufficient sample size of participants and; Additional analysis using data mining techniques will be performed on intervention prescription information in clinical studies collected according to rigorous criteria. On this basis, we will provide a more condensed hypothesis for optimal IM combination therapy by identifying core candidate materials for optimal treatment and discussing the mode of action in detail.

We believe that this study will provide useful knowledge on managing inflammatory skin lesions of psoriasis vulgaris using IM using EAHM. Moreover, it is the goal of the authors to explore whether the information obtained in the process of performing a well-designed meta-analysis for clinical research can contribute to drug discovery.

## Acknowledgments

We would like to thank Editage (www.editage.co.kr) for English language editing.

## Author contributions

**Conceptualization:** Hyehwa Kim, Hee-Geun Jo, Ji-Hye Hwang, Donghun Lee.

**Data curation:** Hyehwa Kim, Hee-Geun Jo.

**Formal analysis:** Hyehwa Kim, Hee-Geun Jo, Ji-Hye Hwang, Donghun Lee.

**Funding acquisition:** Ji-Hye Hwang, Donghun Lee.

**Investigation:** Hyehwa Kim, Hee-Geun Jo, Ji-Hye Hwang, Donghun Lee.

**Methodology:** Hyehwa Kim, Hee-Geun Jo, Ji-Hye Hwang, Donghun Lee.

**Project administration:** Ji-Hye Hwang, Donghun Lee.

**Resources:** Hyehwa Kim, Hee-Geun Jo.

**Software:** Hee-Geun Jo.

**Validation:** Hyehwa Kim, Hee-Geun Jo, Ji-Hye Hwang, Donghun Lee.

**Visualization:** Hee-Geun Jo.

**Writing – original draft:** Hyehwa Kim, Hee-Geun Jo.

**Writing – review & editing:** Hyehwa Kim, Hee-Geun Jo.
